# Generation of two human iPSC lines from patients with autosomal dominant retinitis pigmentosa (UCLi014-A) and autosomal recessive Leber congenital amaurosis (UCLi015-A), associated with *RDH12* variants

**DOI:** 10.1016/j.scr.2021.102449

**Published:** 2021-07

**Authors:** Hajrah Sarkar, Cécile Méjécase, Philippa Harding, Jonathan Eintracht, Lyes Toualbi, Dulce Lima Cunha, Mariya Moosajee

**Affiliations:** a UCL Institute of Ophthalmology, London, UK; b The Francis Crick Institute, London, UK; c Moorfields Eye Hospital NHS Foundation Trust, London, UK; d Great Ormond Street Hospital for Children, NHS Foundation Trust, London, UK

## Abstract

Induced pluripotent stem cell (iPSC) lines were generated from two patients with *RDH12* variants. UCLi014-A is from a patient with heterozygous frameshift mutation c.759del p.(Phe254Leufs*24), associated with autosomal dominant retinitis pigmentosa. UCLi015-A is from a patient with homozygous missense mutation c.619A > G p.(Asn207Asp), associated with Leber congenital amaurosis. Fibroblasts were derived from skin biopsies and reprogrammed using integration free episomal reprogramming plasmids. The iPSC lines expressed pluripotency markers, exhibited differentiation potential *in vitro* and displayed normal karyotypes. These cell lines will act as a tool for disease modelling, enabling comparison of disease mechanisms, identification of therapeutic targets and drug screening.

## 1 Resource table

**Table:** 

Unique stem cell lines identifier	Unique cell line name 1 - UCLi014-A Unique cell line name 2 - UCLi015-A
Alternative names of stem cell lines	Optional name from cell line 1 - RDH12 AD Optional name from cell line 2 - RDH12 AR
Institution	UCL Institute of Ophthalmology
Contact information of distributor	Mariya Moosajee (m.moosajee@ucl.ac.uk)
Type of cell lines	iPSC
Origin	Human
Cell Source	Fibroblasts
Clonality	Clonal
Method of reprogramming	Episomal plasmid
Multiline rationale	Mutations in the same gene
Gene modification	No
Type of modification	N/A
Associated disease	UCLi014-A – Autosomal dominant retinitis pigmentosa UCLi015-A – Leber congenital amaurosis
Gene/locus	Gene: *RDH12* Locus: 14q24.1 Mutation UCLi014-A: NM_152443.2c.759del Mutation UCLi015-A: NM_152443.2c.619A > G
Method of modification	N/A
Name of transgene or resistance	N/A
Inducible/constitutive system	N/A
Date archived/stock date	N/A
Cell line repository/bank	N/A
Ethical approval	11/LO/243 NRES study of congenital eye diseases

## 2 Resource utility

Autosomal dominant variants in *RDH12* are associated with mild retinitis pigmentosa, and autosomal recessive variants are associated with Leber congenital amaurosis. The iPSC lines generated can be used to create disease models, enabling comparison of disease mechanisms between the two conditions and identification of therapeutic targets.

## 3 Resource details

Variants in the retinol dehydrogenase 12 (*RDH12)* gene are commonly associated with Leber congenital amaurosis (LCA), a severe retinal dystrophy characterised by night blindness, nystagmus and central loss of vision in early childhood, eventually leading to complete blindness in adulthood (Fahim et al., 2019). However, in rare cases, heterozygous variants in *RDH12* have been associated with an autosomal dominant late onset mild retinitis pigmentosa phenotype, characterised by nyctalopia and visual field loss, but relatively preserved central vision (Fingert et al., 2008, Sarkar et al., 2020). RDH12 is an NADPH-dependent retinal reductase, expressed in the inner segments of photoreceptors. Loss of functional RDH12 is thought to result in build-up of toxic retinoids, although the exact disease mechanisms are not yet fully understood (Sarkar and Moosajee, 2019). Induced pluripotent stem cells (iPSCs) provide a useful resource to investigate inherited retinal dystrophies in cell types that would otherwise be inaccessible for study. iPSCs derived from patients with *RDH12* variants can be used to create retinal organoids to study the differences in disease mechanisms between autosomal dominant and autosomal recessive mutations. Understanding the molecular pathogenesis of *RDH12*-related retinopathies will enable the identification of therapeutic targets and development of novel therapies.

**Fig. 1:**
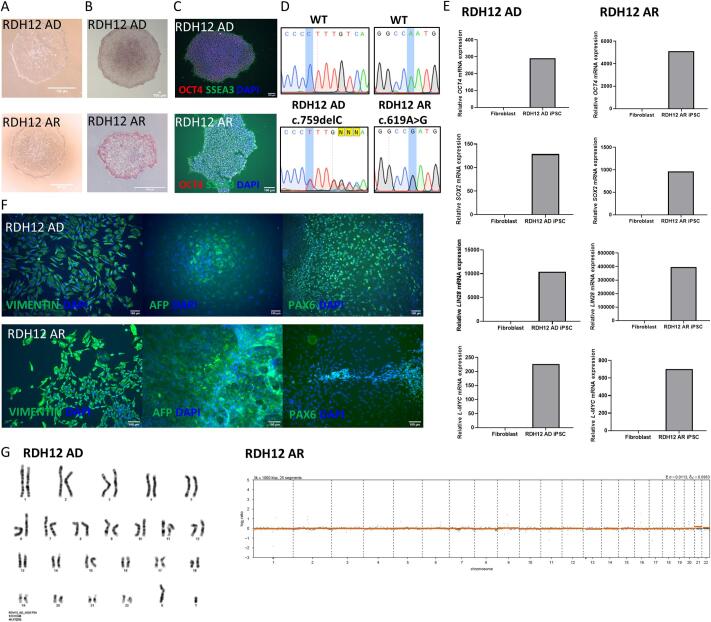

Two iPSC lines were generated from patients with mutations in *RDH12*. The first (UCLi014-A) is from a 32-year old male with autosomal dominant retinitis pigmentosa, carrying a heterozygous frameshift mutation c.759del p.(Phe254Leufs*24). This variant is predicted to result in premature termination and expression of a truncated protein. The second (UCLi015-A) is from a 40 year old female with Leber congenital amaurosis, carrying a homozygous missense mutation c.619A > G p.(Asn207Asp). Fibroblasts were reprogrammed into iPSCs using non-integrating episomal plasmids encoding the reprogramming factors *OCT4*, *KLF4*, *SOX2*, *L-MYC* and *LIN28*. Stem cell-like colonies were picked, and three iPSC clones were expanded and characterised for pluripotency. Mutations were confirmed in iPSCs by Sanger sequencing (Fig. 1D). The morphology of colonies were examined for characteristics of iPSCs, including flat, compact colonies with a cobblestone appearance and large nuclei to cytoplasmic ratio (Fig. 1A). Colonies stained red for alkaline phosphatase, indicating cells are undifferentiated (Fig. 1B). Colonies stained positive for pluripotency markers, OCT4 and SSEA3 (Fig. 1C). Expression of pluripotency markers *OCT4*, *SOX2*, *L-MYC* and *LIN28* were validated using qRT-PCR analysis, which showed upregulation of these markers compared to fibroblast controls (Fig. 1E). G-banding karyotyping revealed a normal male 46,XY karyotype for UCLi014-A and low-pass whole genome sequencing analysis revealed normal female 46,XX karyotype for UCLi015-A (Fig. 1G). Random differentiation of embryoid bodies stained positive for markers of endoderm (AFP), mesoderm (Vimentin) and ectoderm (PAX6), confirming differentiation potential to the three germ layers (Fig. 1F). iPSC identity was confirmed by STR analysis (Table S2). Absence of mycoplasma was confirmed in iPSCs (Table S3).

In conclusion, two human iPSCs lines were generated from patients with *RDH12*-related retinopathies. These iPSC lines provide a valuable resource for disease modelling, comparison of disease mechanisms, therapeutic target identification and drug screening.

## 4 Materials and methods

### 4.1 Fibroblast derivation and culture

Skin biopsies were placed in 400 μL digestion media (DMEM high glucose, GlutaMAX Supplement, pyruvate, 20% FBS, 0.25% Collagenase I, 0.05% DNase I, Pen/strep), incubated overnight at 37˚C, 5% CO_2_, then plated in derivation media (DMEM, 20% FBS and Pen/Strep). Fibroblasts were cultured in fibroblast media (DMEM, 10% FBS and Pen/Strep) and passaged with TrypLE Express (Gibco) (See Table 1).

**Table 1:** Summary of lines.

iPSC line names	Abbreviation in figures	Gender	Age	Ethnicity	Genotype of locus	Disease
RDH12 AD (UCLi014-A)	RDH12 AD	Male	32	Israeli Kurdistan and Tunisian	N/A	Retinitis pigmentosa
RDH12 AR (UCLi015-A)	RDH12 AR	Female	40	Pakistani	N/A	Leber congenital amaurosis

### 4.2 Validation of mutation

DNA was extracted using QIAamp DNA Micro Kit (Qiagen). *RDH12* was amplified using MyTaq PCR (Bioline) (Table 3). Mutations were confirmed by Sanger sequencing.

### 4.3 Fibroblast reprogramming and iPSC culture

1 × 10^6^ fibroblast cells were electroporated with 1 μg of each episomal plasmid (Table S1) using Neon Transfection System (1700 V, 20 ms, 1 pulse). Cells were plated into 1 well of a Matrigel-coated (Corning) 6-well plate in fibroblast media. On day 5, medium was changed to 3:1 fibroblast medium:mTeSR Plus (Stemcell). On day 7, medium was changed to 1:1 fibroblast medium:mTeSR Plus, from day 9 medium was changed daily with mTESR Plus. Colonies were expanded manually up to passage 4, then passaged using ReLeSR (Stemcell) at a 1:10 split ratio. iPSCs under passage 15 were used for all further characterisations (See Table 2).

**Table 2:** Characterization and validation.

Classification	Test	Result	Data
Morphology	Photography	Normal	Fig. 1 panel A
Phenotype	Qualitative analysis: Immunocytochemistry	Positive for pluripotency markers OCT4 and SSEA3	Fig. 1 panel C
	Qualitative analysis: Alkaline phosphatase activity	Visible activity	Fig. 1 panel B
	Quantitative analysis: qRT-PCR	Expression of *OCT4, SOX2, L-MYC* and *LIN28*	Fig. 1 panel E
Genotype	Karyotype (G-banding) and resolution	RDH12 AD − 46XY Resolution 400	Fig. 1 panel G
	Low-pass whole genome	RDH12 AR – 46XX	Fig. 1 panel G
Identity	Microsatellite PCR (mPCR)	N/A	N/A
	STR analysis	16 STR analyzed, all matched	Supplementary Table 2
Mutation analysis (IF APPLICABLE)	Sequencing	RDH12 AD - Heterozygous frameshift mutation c.759del p.(Phe254Leufs*24) RDH12 AR – Homozygous missense mutation c.619A > G p.(Asn207Asp)	Fig. 1 panel D
	Southern Blot OR WGS	N/A	N/A
Microbiology and virology	Mycoplasma	Mycoplasma testing by MycoAlert^TM^ Mycoplasma Detection Kit (Lonza): Negative	Supplementary Table 3
Differentiation potential	e.g. Embryoid body formation	Positive for three germ layer markers: endoderm marker AFP, mesoderm marker Vimentin and ectoderm marker PAX6	Fig. 1 panel F
Donor screening (OPTIONAL)	HIV 1 + 2 Hepatitis B, Hepatitis C	N/A	N/A
Genotype additional info (OPTIONAL)	Blood group genotyping	N/A	N/A
	HLA tissue typing	N/A	N/A

### 4.4 Alkaline phosphatase staining

Cells were stained using StemAb Alkaline Phosphatase Staining Kit II (Reprocell).

### 4.5 Immunocytochemistry

Cells were fixed using 4% PFA for 20 min at 4 °C, permeabilised and blocked for 1 h at room temperature (RT) in 10% normal goat serum (NGS), 0.1% X-100, PBS. Cells were incubated for 1 h with primary antibodies diluted in 1% NGS at RT (Table 3). Secondary antibodies and DAPI were added for 1 h at RT. Cells were washed and imaged using the EVOS M7000 Imaging System.

**Table 3:** Reagents details.

Antibodies used for immunocytochemistry			
	Antibody	Dilution	Company Cat # and RRID
Pluripotency Markers	Mouse anti-OCT4	1:100	Santa Cruz Biotechnology Cat# sc-5279, RRID:AB_628051
	Rat anti-SSEA3	1:50	Millipore Cat# MAB4303, RRID:AB_177628
Differentiation Markers	Mouse anti-AFP	1:300	Santa Cruz Biotechnology Cat# sc-51506, RRID:AB_626514
	Mouse anti-Vimentin	1:250	Santa Cruz Biotechnology Cat# sc-6260, RRID:AB_628437
	Rabbit anti-PAX6	1:100	Covance Cat# PRB-278P, RRID:AB_291612
Secondary antibodies	Goat anti-Mouse IgG (H + L) Cross-Adsorbed Secondary Antibody, Alexa Fluor 647	1:400	Thermo Fisher Scientific Cat# A-21235, RRID:AB_2535804
	Goat anti-Rat IgG (H + L) Highly Cross-Adsorbed Secondary Antibody, Alexa Fluor 488	1:400	Thermo Fisher Scientific Cat# A-11006, RRID:AB_2534074
	Goat anti-Rabbit IgG (H + L) Highly Cross-Adsorbed Secondary Antibody, Alexa Fluor 488	1:400	Thermo Fisher Scientific Cat# A32731, RRID:AB_2633280
	Goat anti-Mouse IgG (H + L) Cross-Adsorbed Secondary Antibody, Alexa Fluor 488	1:400	Thermo Fisher Scientific Cat# A-10011, RRID:AB_2534069

Primers			
	Target	Forward/Reverse primer (5′-3′)	
Pluripotency Markers (qRT-PCR)	OCT4	CCCCAGGGCCCCATTTTGGTACC/ACCTCAGTTTGAATGCATGGGAGAGC	
	SOX2	TTCACATGTCCCAGCACTACCAGA/TCACATGTGTGAGAGGGGCAGTGTGC	
	LIN28	AGCCATATGGTAGCCTCATGTCCGC/TCAATTCTGTGCCTCCGGGAGCAGGGTAGG	
	L-MYC	GCGAACCCAAGACCCAGGCCTGCTCC/CAGGGGGTCTGCTCGCACCGTGATG	
House-Keeping Genes (qRT-PCR)	GAPDH	ACAGTTGCCATGTAGACC/TTTTTGGTTGAGCACAGG	
Targeted mutation sequencing (Sanger)	RDH12 exon 8	TGGCCAGGAGTGGTACCTGC/GCAACTCTTCCCAACACATA	
	RDH12 exon 7	GACCATTAGAGTTACTCATGGC/CGTGCATGTTTGACAGCCTG	

### 4.6 qRT-PCR

RNA was extracted using RNeasy Mini Kit (Qiagen). cDNA was synthesised from 1 μg of RNA using Superscript II First Strand cDNA synthesis kit (Invitrogen). Transcript levels were analysed using SYBR Green MasterMix on StepOne Plus RealTime PCR System (Table 3). Relative expression of each target gene was normalised to *GAPDH* and compared to fibroblast expression.

### 4.7 Embryoid body mediated spontaneous differentiation

Embryoid bodies (EBs) were formed by dissociation of cells using ReLeSR and culturing in Aggrewell media (Stemcell) supplemented with 10 µM Y27632 for 10 days. EBs were plated in 0.1% gelatin-coated plates in DMEM/20% FBS for 11 days, where EBs attached and spontaneously differentiated. Cells were fixed and immunostained for germ layer markers AFP (endoderm), Vimentin (mesoderm) and marker PAX6 (ectoderm) (Table 3).

### 4.8 Karyotyping

iPSCs were sent to Cell Guidance Systems for karyotyping and 20 metaphases were counted.

### 4.9 Low-pass whole genome sequencing and STR analysis

DNA was extracted using QIAamp DNA Micro Kit (Qiagen). For low-pass WGS, libraries were produced using Illumina DNA Prep library prep kit and sequenced on Illumina HiSeq 4000 with paired 100 bp reads. After alignment, copy number estimation was performed using the QDNASeq package (Scheinin et al., 2014). Short Tandem Repeat (STR) profiling was obtained for each cell line with Promega PowerPlex16HS system and was compared back to any available on commercial cell banks.

### 4.10 Mycoplasma testing

Absence of mycoplasma contamination was confirmed using MycoAlert^TM^ Mycoplasma Detection Kit (Lonza).

## Declaration of Competing Interest

The authors declare that they have no known competing financial interests or personal relationships that could have appeared to influence the work reported in this paper.
