# Supplementary material for: Generation of two human iPSC lines from patients with autosomal dominant retinitis pigmentosa (UCLi014-A) and autosomal recessive Leber congenital amaurosis (UCLi015-A), associated with RDH12 variants
Source: Stem Cell Res. 2021 Jul;54:102449. doi: 10.1016/j.scr.2021.102449 (PMC8363920; doi:10.1016/j.scr.2021.102449)
Supplement: Supplementary data 1 [file mmc1.docx]

**Supplementary files**

Supplementary Table 1 - Addgene Episomal Vectors for Reprogramming.

| **Episomal Plasmid** | **Addgene ID#** | **Encodes** |
| --- | --- | --- |
| **pCXLE-hSK** | 27078 | SOX2 and KLF4 |
| **pCXLE-hUL** | 27080 | L-MYC and LIN28 |
| **pCXLE-hOCT3/4-shp53-F** | 27077 | OCT3/4 and shRNA against p53 |
| **pCXWB-EBNA1** | 37624 | transient EBNA-1 |

Supplementary Table 2 – STR analysis

| **STR site** | **RDH12 AD Fibroblast** | **RDH12 AD iPSC** | **RDH12 AR Fibroblast** | **RDH12 AR iPSC** |
| --- | --- | --- | --- | --- |
| **FGA** | 19,23 | 19,23 | 21,27 | 21,27 |
| **TPOX** | 9,10 | 9,10 | 8,11 | 8,11 |
| **D8S1179** | 10,12 | 10,12 | 12,15 | 12,15 |
| **vWA** | 16,18 | 16,18 | 14,16 | 14,16 |
| **AMEL** | X,Y | X,Y | X,X | X,X |
| **Penta D** | 12,14 | 12,14 | 9,11 | 9,11 |
| **CSF1PO** | 11,12 | 11,12 | 10,12 | 10,12 |
| **D16S539** | 8,9 | 8,9 | 11,13 | 11,13 |
| **D7S820** | 12,12 | 12,12 | 10,11 | 10,11 |
| **D13S317** | 11,12 | 11,12 | 12,12 | 12,12 |
| **D5S818** | 12,13 | 12,13 | 12,12 | 12,12 |
| **Penta E** | 13,18 | 13,18 | 7,11 | 7,11 |
| **D18S51** | 13,13 | 13,13 | 13,16 | 13,16 |
| **D21S11** | 28,30.2 | 28,30.2 | 28,31.2 | 28,31.2 |
| **TH01** | 6,7 | 6,7 | 7,7 | 7,7 |
| **D3S1358** | 16,16 | 16,16 | 16,16 | 16,16 |

Supplementary Table 3 – MycoAlert^TM^ Mycoplasma Detection Kit (Lonza). Ratio <1.2 – negative.

| **Sample** | **Reading A** | **Reading B** | **Ratio** |
| --- | --- | --- | --- |
| **Water Control** | 10653 | 1495 | 0.140336 |
| **RDH12 AD** | 11434 | 6049 | 0.529036 |
| **RDH12 AR** | 9223 | 5693 | 0.617261 |
